# A global, descriptive analysis of the sources used to regulate abortion care

**DOI:** 10.1080/26410397.2026.2710920

**Published:** 2026-08-07

**Authors:** Sanhita Ambast, Hazal Atay, Antonella Lavelanet

**Affiliations:** a Consultant, UNDP-UNFPA-UNICEF-WHO-World Bank Special Programme of Research, Development and Research Training in Human Reproduction (HRP), Department of Sexual, Maternal, Child, Adolescent Health and Ageing, World Health Organization, London, UK. *Correspondence*: Sanhitaa@gmail.com; b Consultant, UNDP-UNFPA-UNICEF-WHO-World Bank Special Programme of Research, Development and Research Training in Human Reproduction (HRP), Department of Sexual, Maternal, Child, Adolescent Health and Ageing, World Health Organization, Paris, France; c Medical Officer, Consultant, UNDP-UNFPA-UNICEF-WHO-World Bank Special Programme of Research, Development and Research Training in Human Reproduction (HRP), Department of Sexual, Maternal, Child, Adolescent Health and Ageing, World Health Organization, Avenue Appia 20, 1211 Geneva, Switzerland

**Keywords:** abortion, sources, regulation, abortion exceptionalism, stigma, criminalisation

## Abstract

An enabling environment is essential for delivering quality, comprehensive abortion care. According to the WHO, abortion should be treated like other healthcare services, regulated by healthcare law, and guided by evidence-based standards and guidelines. This paper examines abortion regulations across 183 countries, drawing on data from the Global Abortion Policies Database, up to May 2025. It divides sources relevant to the regulation of abortion care into seven categories: Criminal/Penal Code, Abortion-specific Law, Health Regulation/Clinical Guidelines, Constitution, Case Law, Essential Medicines List /Registered List, and other sources. The findings of this paper indicate that abortion is regulated through an often complex and fragmented framework, characterised by over-reliance on the general criminal law, the use of multiple and sometimes contradictory sources, and information gaps in certain key areas. 169 countries regulate abortion through their Criminal or Penal Code, with general criminal law the only source regulating abortion in 27 countries. Information about grounds and gestational limits for abortion can be found in the general criminal law in 123 countries. 46 countries have abortion-specific laws; 16 countries include relevant provisions in their constitution; in 90 countries, abortion-related information is spread across five or more source documents; and the status of conscientious objection is not regulated by any document in 109 countries. This landscape risks exacerbating existing barriers to abortion care, including stigma and inadequate access to information, and reinforces abortion exceptionalism. These findings raise questions about how reforming the regulation of abortion, including the nature and number of source documents involved, can shape an enabling environment for abortion care. They also highlight the need to identify effective strategies for safeguarding the health and rights of people seeking abortions. DOI: 10.1080/26410397.2026.2710920

## Plain language summary

An enabling environment is essential for delivering quality, comprehensive abortion care. The WHO says abortion should be treated like other healthcare services. It should be regulated by healthcare law, evidence-based standards and guidelines. We used information from the Global Abortion Policies Database to examine abortion regulations across 183 countries up to May 2025. We looked at the type and number of sources regulating aspects of abortion care. We found that abortion is regulated through an often complex and fragmented framework. This is characterised by over-reliance on the general criminal law. There are many, sometimes contradictory, sources, and information gaps in certain key areas. Most countries regulate abortion through their Criminal or Penal Code. General criminal law is the only source regulating abortion in 27 countries. 46 countries have laws specifically about abortion. 16 countries include relevant provisions in their constitution. Information related to abortion is spread across five or more source documents in 90 countries. The status of conscientious objection is not regulated by any document in 109 countries. This landscape risks making existing barriers – like stigma or lack of information about abortion care – worse. It also reinforces treating abortion as an exceptional form of healthcare. These findings raise questions about how reform of abortion regulation could make the environment for abortion care more enabling. They highlight the need to find effective ways to safeguard the health and rights of people seeking abortions.

## Résumé

Un environnement favorable est essentiel pour assurer des soins liés à l’avortement complets et de qualité. Selon l’OMS, l’avortement devrait être traité comme d’autres interventions de santé et être encadré par le droit de la santé ainsi que des normes et directives fondées sur des bases factuelles. Cet article examine la réglementation de l’avortement dans 183 pays à partir d’informations tirées de la base de données mondiale sur les politiques en matière d’avortement (*Global Abortion Policies Database*), jusqu’en mai 2025. Il classe les sources pertinentes pour la réglementation des soins liés à l’avortement en sept catégories: code pénal, lois spécifiques à l’avortement, règlement sanitaire/guides de pratique clinique, Constitution, jurisprudence, listes/registres de médicaments essentiels et autres sources. Les conclusions de cet article indiquent que l’avortement est réglementé par un cadre souvent complexe et fragmenté, caractérisé par une dépendance excessive au droit pénal général, le recours à des sources multiples et parfois contradictoires, et des lacunes d’information dans certains domaines clés. Cent soixante-neuf pays réglementent l’avortement par le biais de leur code pénal, le droit pénal général étant la seule source de réglementation de l’avortement dans 27 pays. Les informations relatives aux motifs et aux limites gestationnelles pour l’avortement figurent dans le droit pénal général de 123 pays. Quarante-six pays disposent de lois spécifiques en matière d’avortement; 16 pays incluent des dispositions pertinentes dans leur Constitution; dans 90 pays, l’information relative à l’avortement est dispersée dans au moins cinq documents de référence; et aucun texte n’encadre le statut de l’objection de conscience dans 109 pays. Cette situation risque d’exacerber les obstacles existants à l’accès aux soins liés à l’avortement, tels que la stigmatisation et l’accès insuffisant à l’information, et elle renforce l’exceptionnalisme de l’avortement. Ces résultats soulèvent des questions quant à la manière dont une réforme de la réglementation de l’avortement, notamment concernant la nature et le nombre de documents de référence, peut mettre en place un environnement favorable à l’accès aux soins. Ils soulignent aussi la nécessité d’identifier des stratégies efficaces pour protéger la santé et les droits des personnes souhaitant avorter.

## Resumen

Un entorno propicio es esencial para la prestación de servicios de atención integral del aborto de calidad. Según la OMS, el aborto debe tratarse como otros servicios de salud, regulado por la ley de atención médica y por normas y directrices basadas en evidencia. Este artículo examina las regulaciones del aborto en 183 países, basándose en datos de la Base de Datos Globales sobre Políticas de Aborto, hasta mayo de 2025. Divide las fuentes pertinentes a la regulación de los servicios de aborto en siete categorías: Código Penal, Ley sobre aborto específicamente, Regulación de salud/Directrices clínicas, Constitución, Jurisprudencia, Lista de medicamentos esenciales/Lista registrada y otras fuentes. Los hallazgos de este artículo indican que el aborto es regulado por medio de un marco a menudo complejo y fragmentado, caracterizado por una dependencia excesiva del derecho penal general, el uso de múltiples fuentes que a veces son contradictorias, y lagunas de información en ciertas áreas clave. En 169 países, el aborto es regulado por medio del Código Penal, y el derecho penal general es la única fuente de regulación del aborto en 27 países. La información sobre las causales y los límites gestacionales para el aborto se encuentra bajo el derecho penal general en 123 países. En 46 países existen leyes sobre aborto específicamente; en 16 países, la Constitución contiene disposiciones pertinentes; en 90 países, la información relacionada con el aborto es difundida entre cinco o más documentos fuente; y en 109 países, el estatus de objetor de conciencia no es regulado por ningún documento. Este panorama corre el riesgo de exacerbar las barreras actuales a los servicios de aborto, tales como estigma y acceso inadecuado a la información, y refuerza el excepcionalismo del aborto. Los hallazgos plantean interrogantes sobre cómo la reforma de la regulación del aborto, que incluye la naturaleza y la cantidad de documentos fuente involucrados, puede guiar un entorno propicio para los servicios de aborto. Además, se destaca la necesidad de identificar estrategias eficaces para salvaguardar la salud y los derechos de las personas que buscan abortos.

## Introduction

The World Health Organization's Abortion Care Guideline emphasises that an enabling environment is essential for delivering quality, comprehensive abortion care.^1^ This environment should reflect a respect for human rights underpinned by a supportive framework of law and policy. Key components include the provision of accurate, accessible information such as where and how to obtain abortion services, associated costs, and relevant legal considerations. As such, a supportive, universally accessible, affordable and well-functioning health system is needed. Fundamentally, the WHO recommends that abortion be governed in the same manner as other healthcare services, guided by general health laws, clinical best practices, professional training, and evidence-based standards.^1^

In general, law can be a powerful tool to safeguard and promote public health, especially when public health laws are evidence-based and fairly and effectively implemented.^2^ The way abortion is regulated, including the legal sources and frameworks applied, shapes the context in which abortion seekers and providers operate.^3^ Scholars have noted how law can be a determinant of health and how the nature of abortion regulation can impact access to care in practice.^4^ For example, it can impact how risk and resources are distributed within the health system,^5^ and “burdensome overregulation” can become a barrier to care.^6^ Under-regulation can also mean that the law and policy framework is inadequate to ensure equitable access to care.^4^

The Abortion Care Guideline notes that, unlike other health services, abortion is commonly regulated to varying degrees through the criminal law, in addition to health legislation.^1^ Key information on abortion regulation can also often be found in case law and constitutional provisions. This further raises the question of abortion exceptionalism, that is, whether abortion is treated differently from other forms of health care in law and policy, programming and medical practice.^7–10^

The nature, type and number of sources involved in the regulation of abortion care can carry important implications beyond their direct legal effect.^5^ These sources serve a signalling function, reflecting how a state conceptualises abortion within its broader legal and policy framework. Furthermore, how certain sources are drafted can influence the creation of an enabling environment for abortion care. The Abortion Care Guidance emphasises that states should adopt and regularly update evidence-based standards for sexual and reproductive health (SRH) services, including abortion, to reflect medical advancements and ensure equitable access.^1^ National standards and clinical guidelines play a critical role in translating this recommendation into practice. When abortion is regulated through health regulations or clinical protocols, it allows for timely updates and alignment with evolving medical evidence. In contrast, other legal sources such as constitutions, general statutes, or case law are often developed by non-medical actors, are less responsive to scientific developments, and are more difficult to revise.^11^

In this context, this paper contributes to the discussion on fostering an enabling environment for abortion care by examining how abortion is currently regulated across countries. Drawing on data from the Global Abortion Policies Database (GAPD), this paper describes the number and range of sources that are pertinent to the regulation of abortion globally. The GAPD was developed as a tool to enhance transparency and promote Member State accountability in protecting health and human rights, and compiles information related to laws, policies, and service delivery for all WHO Member States. It reflects country-specific information on a broad range of policy issues relevant to abortion regulation, linked to source documents that can be accessed and downloaded. The methodology for how the GAPD is coded is detailed elsewhere.^12^ Other papers have used the GAPD to analyse criminal penalties for abortion,^13^ legal categories for abortion,^14^ and financial coverage for abortion care across countries.^15^ This paper expands on that work by focusing on a dimension of abortion regulation that has not yet been systematically studied: the breadth and diversity of the policy sources on which national abortion frameworks are built.

## Methods

For the purposes of this paper, we define a “source” as any document that contains relevant information to the regulation of abortion within a country. We draw on data from the Global Abortion Policies Database (GAPD), as available up to May 2025, to analyse the number and types of source documents that countries use to regulate abortion. The GAPD contains official government sources and government-endorsed documents. The GAPD is populated using data that is publicly available in each country. Legal and policy sources that exist but are not easily accessible may not be reflected on the GAPD. Sources are periodically updated as and when they are identified.

This paper focuses on identifying the existence and classification of these sources, without evaluating their content or legal impact. As the GAPD is regularly updated to reflect newly available or identified documents, recent legal reforms may not yet be captured in the dataset. All the data used in this paper, including the regulatory sources identified for each country, can be accessed, and downloaded from the GAPD.^16^

The analysis includes 183 countries, limited to WHO member states. We excluded two countries from the analysis (Democratic People's Republic of Korea and Marshall Islands) due to the absence of any publicly available data on abortion regulation. We excluded an additional nine countries (Australia, Bosnia and Herzegovina, Canada, China, Mexico, Nigeria, Switzerland, the United Kingdom of Great Britain and Northern Ireland, and the United States of America) because the regulation of abortion varies within sub-national jurisdictions in the country. While these contexts fall outside the scope of this analysis, their diverse policy environments underscore the broader variability in regulatory approaches globally.

The GAPD captures both the specific document and source type, systematically organised into 15 categories, including, for example, a Reproductive Health Act, General Medical Health Act, Constitution, Criminal/Penal Code, Civil Code, Ministerial Order/Decree, Case Law, and Clinical Guidelines. For the purposes of this analysis, we have consolidated these into 7 categories:

1. Criminal/Penal Code
2. Abortion-specific Law
3. Health Regulation/Clinical Guidelines
4. Constitution
5. Case Law
6. Essential Medicines List (EML)/Registered List
7. Other sources

(Other sources include Reproductive Health Act, General Medical Health Act, Civil Code, Ministerial Order/Decree, Law on Medical Practitioners, Law on Funding, and Law on Health Care Services, and a Medical Ethics Code.) This grouping allows for a more manageable and comparative analysis while preserving the diversity of legal instruments used across jurisdictions.

This paper also examines key areas of abortion regulation addressed by the source documents. The GAPD organises abortion-related information into five broad categories: (1) legal grounds and gestational limits, (2) additional requirements to access safe abortion, (3) clinical and service delivery aspects of abortion, (4) conscientious objection, and (5) penalties for abortion. For the purposes of this analysis, we adopt these five categories with one modification. In the GAPD, aspects of the regulation of abortion medicines (mifepristone and/or misoprostol) are included under “clinical and service delivery aspects of abortion”. However, because this aspect is typically governed through a country's EML or register of approved medicines and is distinct from other clinical or service delivery regulations, we treat it as a separate, sixth category. This distinction avoids the misleading impression that drug registration documents apply broadly to all clinical aspects of abortion care. Accordingly, this paper analyses the types of sources associated with six categories of abortion-related regulation.

Each category encompasses multiple elements that reflect different dimensions of regulation. For example, the broad category “clinical and service delivery aspects of abortion” includes information on who can provide services, where they can be provided, and what methods of abortion are available in a particular country. In many cases, multiple sources contain information relevant to the same category. For example, a country's Constitution, Penal Code, and Health Guidelines may all address legal grounds and gestational limits, and each would be recorded as a relevant source. A source was included in the analysis for a given regulatory category if it addressed at least one component within a broader category.

Countries were deemed to regulate a category if at least one sub-question within the category was addressed in an official source. For example, if a source specified who can perform abortions but provided no information on where or how they are performed, the country was still marked as having information for clinical and service delivery.

On the GAPD, the label “not specified” is used against a data point when a source exists and would be expected to contain relevant information, but that information is missing. The term “no data found” is applied when no relevant source can be identified for a given issue. For the purposes of this analysis, we have merged these two categories. We used a standardised extraction form to collect information about the source documents listed in the GAPD. One author conducted the initial data extraction (SA); a second author (HA) cross-checked the data. Any discrepancies were reviewed and discussed with a third reviewer (AL). Where the number of countries in any category of analysis is under ten, we have listed them all in text.

Within the GAPD, countries are grouped by regional groups: Africa, Asia, Europe, Latin America, North America and Oceania. Canada and the United States are excluded from this paper due to the sub-national variation in abortion regulation. As North America comprises only these two countries, the region is excluded entirely from the analysis and does not appear in the graphs.

We analyse information reflected in the GAPD and are reliant on the methodology and limitations of the GAPD. The GAPD is updated at the point at which a new source becomes known or available, which means there may be reform not currently reflected on the site, and therefore is not part of this study. Similarly, while the GAPD includes case law; this is not updated often enough to reflect the complex, differing and rapidly changing legal environment in many countries. The completeness of the GAPD is also limited by the accessibility of source materials and the feasibility of translating them into usable formats. Finally, the GAPD does not reflect information about how laws and policies are implemented, or whether they effectively translate into access to abortion care in practice.

## Results

### Range and numbers of sources

Abortion-related information is distributed across a variety of legal and policy documents globally, which serve as instruments of regulation. A total of 169 countries regulate abortion through their Criminal or Penal Code. A total of 157 countries include abortion-related provisions in an EML / registered list of drugs. A total of 124 countries reference abortion in other types of sources, such as civil codes, ministerial decrees, or medical ethics codes. A total of 98 countries use Health Regulations or Clinical Guidelines. A total of 46 countries have abortion-specific laws. A total of 16 countries include relevant provisions in their constitution, and 15 countries reference abortion in case law.

Graph 1 provides details by region about the variety of source types:

**Graph 1.**
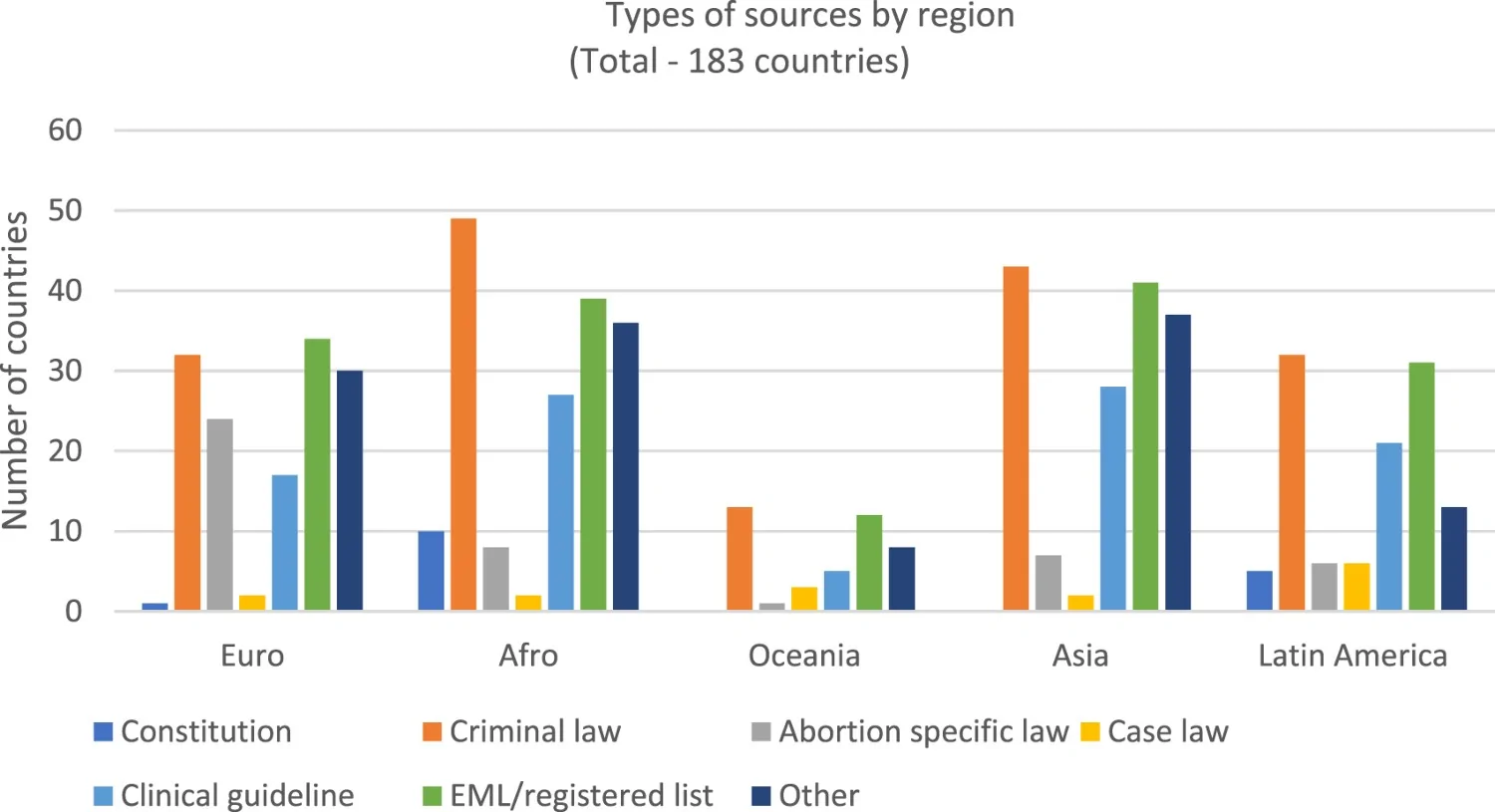
Types of sources by WHO region

93 countries regulate abortion through fewer than five sources. However, in 90 countries, abortion-related information is spread across five or more documents. Among these, 19 countries have 10 or more relevant documents. Only three countries (Bahamas, San Marino, Sao Tome and Principe) have a single document that regulates abortion.

In eight countries (Angola, Bolivia, Honduras, Mozambique, Nepal, Oman, Senegal and Uganda), there is a conflict between two or more source documents regarding how certain aspects of abortion should be regulated. For example, in Nepal, according to the Penal Code, in cases of incest, pregnancies can be terminated until 18 weeks. However, the National Comprehensive Abortion Care Guidelines and the Right to Safe Motherhood and Reproductive Health Act suggest that the gestational limit in cases of incest is 28 weeks. In Oman, the Penal Code does not indicate any grounds for abortion. However, the Law Regulating the Practice of the Medical Profession stipulates that abortion can be carried out in cases of foetal impairment. Similarly, in Senegal, while the Penal Code does not include a life ground, a Medical Code of Ethics document states that a therapeutic abortion can be performed if this intervention is the only way to safeguard the life of the mother.

### Sources used to regulate key aspects of abortion care

This section outlines the types of legal and policy documents that serve as sources of information on the six broad categories of abortion-related regulation defined in this paper (see Graph 2).

**Graph 2.**
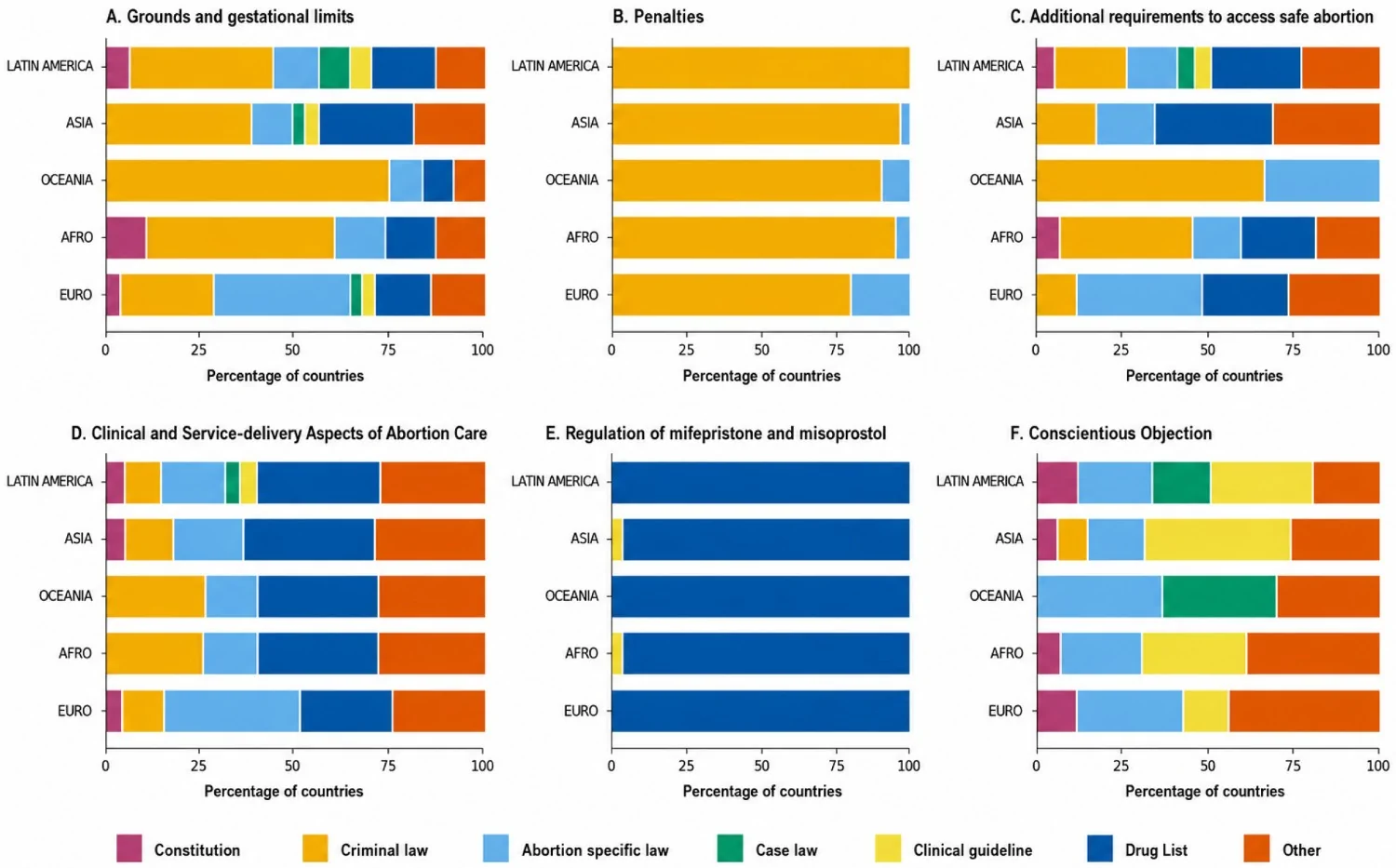
Legal sources governing abortion care by WHO Regions

Information about grounds and gestational limits for abortion can be found in the general criminal law in 123 countries; in other types of sources in 66 countries; in health regulations / clinical guidelines in 50 countries; in an abortion-specific law in 42 countries; in the constitution in 11 countries; and in case law in 7 countries (Argentina, Brazil, Colombia, Ecuador, India, South Korea and Poland).

Information about the additional requirements to access safe abortions is contained in other types of sources in 72 countries; in health regulations / clinical guidelines in 54 countries; the general criminal law in 53 countries; in an abortion specific law in 42 countries; in the constitution in 3 countries (Bolivia, Eswatini, and Kenya); and in case law in 1 country (Colombia).

Information about clinical and service-delivery aspects of abortion care can be found in health regulations / clinical guidelines in 82 countries; in other types of sources in 67 countries; in an abortion-specific law in 41 countries; in the general criminal law in 35 countries; in the constitution in 1 country (France); in case law in 1 country (Colombia).

The use of mifepristone and misoprostol is regulated through an EML / registered list of drugs in 168 countries, and through clinical guidelines in 2 countries (Bangladesh and Malawi).

Conscientious objection is regulated through other types of sources in 35 countries; through an abortion specific law in 24 countries; through clinical guidelines in 21 countries through the general criminal law in 5 countries (Austria, Belize, Israel, Monaco and Panama); through case law in 4 countries (Argentina, Chile, Colombia, and New Zealand); and through the constitution in 2 countries (France and Kenya).

Penalties for abortion are regulated through the general criminal law in 164 countries; through an abortion specific law in 10 countries; and through other types of laws in 3 countries (Denmark, France, and Saudi Arabia).

### Gaps in information and regulation

We also identified gaps in information related to the regulation of abortion-related care, as reflected on the GAPD. In 18 countries, abortion is legal in some circumstances, but no document was found to provide information on additional requirements to access safe abortion. In 17 countries, abortion is legal in some circumstances, yet no document was found to provide information on the clinical and service-delivery aspects of abortion care. These gaps were distributed across regions, with a relatively even spread, except in the European region where no such gaps were identified. While 74 countries regulate conscientious objection in some way, the status of conscientious objection is not regulated by any document in 109 countries.

### Use of the general criminal law

In 27 countries, the general criminal law functions as the sole source regulating aspects of abortions. This includes countries where abortion is regulated through only one document (i.e. the general criminal law) or where only two sources of abortion regulation are available (i.e. the general criminal law and an EML / registered list of drugs).

In 14 countries, the general criminal law is not used at all in the regulation of abortion. In these cases, relevant provisions are instead found in other legal or policy documents, such as reproductive health laws and abortion-specific laws.

Graph 3 provides details by region about how the general criminal law is used:

**Graph 3.**
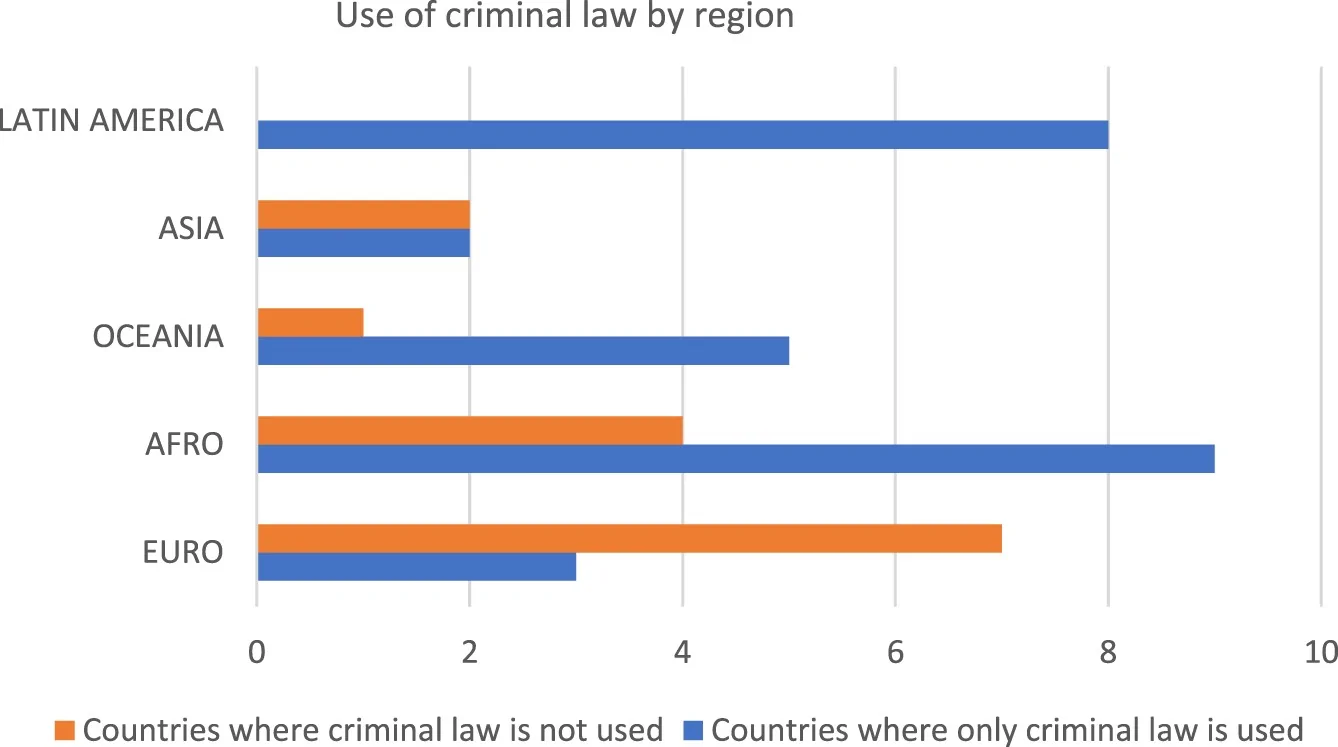
How the general criminal law is used by region

## Discussion

The findings of this paper highlight critical patterns in how abortion care is regulated globally. They also raise questions around how the constellation of source documents relevant to the regulation of abortion care may impact the rights of abortion seekers and providers, including whether individuals can access accurate and consistent information and the extent to which care can be obtained without stigma.

### Criminal law and stigma

Despite the World Health Organization's 2022 Abortion Care Guideline recommending that abortion be regulated similarly to other health services, through evidence-based health law and policy, the data show that the general criminal code remains the primary regulatory instrument in most countries and across multiple aspects of abortion care. The WHO has noted that criminalisation can negatively affect the rights of pregnant individuals and may deter the provision of quality care.^1^

Reflecting the global extent of such criminalisation, a recent analysis reported that as of 2023, 134 countries penalised abortion-seekers, 181 countries penalised abortion-providers and 159 countries penalised persons assisting in abortions.^12^ While the maximum penalty was between 0 and 5 years of imprisonment in a majority of countries, it was much higher in some others, with life imprisonment being a possibility in six countries. Some countries prescribed fines, and professional sanctions for providers and those who assist where abortion is unlawful.

The negative consequences of criminalising abortion are well documented.^17,18^ The association of abortion with criminal offences discourages individuals from seeking care and can delay access to care, increase costs for abortion seekers, and create barriers to access, even where abortion is legally permitted.^19–24^ By framing abortion as a criminal matter, the law may compound the stigma abortion seekers and providers experience, making people more hesitant to provide abortions, and increasing the workload of abortion providers.^25–28^ This further raises the question of whether the continued reliance on criminal law to regulate abortion contributes to reinforcing the stigma surrounding abortion.

The reliance on the general criminal code places an undue burden on both abortion seekers and providers to navigate complex and often opaque legal texts to access or deliver care. It also creates an expectation of legal literacy and clarity that may not align with real-world healthcare settings, leading to confusion, delays, and legal risk even where abortion is permitted.^29,30^

### Concerns around access to information

Our findings raise important questions about whether fragmented regulation and reliance on multiple, and sometimes contradictory, legal sources hinder access to accurate and timely information about abortion care. These concerns are amplified by the lack of publicly available guidance on how key aspects of abortion services are regulated in some countries. According to the WHO's 2022 Abortion Care Guideline, ensuring that individuals can access relevant, accurate and evidence-based health information is a critical step in improving access to and quality of abortion services.^1^ Furthermore, human rights standards reinforce this obligation, requiring that states ensure the provision of comprehensive, non-discriminatory, scientifically accurate and age-appropriate education on sexuality and reproduction, including information on abortion.^31–33^ Yet, studies consistently show that people seeking abortions often lack accurate information about when, where and how services are available.^34–36^ A lack of adequate information, coupled with fear of violating laws, can act as a barrier for people seeking abortions.^37^ Providers, too, may be uncertain about the legal framework governing abortion, which can lead to reluctance in providing these services even where legal.^29,30^ Where regulations are unclear or vague, providers rely on their personal values and interpretations, which can be a barrier to care.^27^ Restrictive and confusing laws can further impact the quality of training providers receive.^38^ These gaps in knowledge, fuelled by complex and inconsistent legal structures, can deter both access to and provision of safe abortion care.

Our findings reveal that in several countries where abortion is legally permitted under certain conditions, there is no publicly available information detailing the clinical and service-delivery aspects of care such as who is authorised to perform abortions, where they can be provided, what methods are permitted, or how services are financed. These gaps may reflect either a lack of public access to relevant documents or an absence of regulation altogether, both of which raise serious concerns for public health and human rights.

As noted in the Methods section, two countries have been excluded from the analysis due to the absence of any publicly available data on abortion regulation, and nine countries have been excluded because the regulation of abortion varies within sub-national jurisdictions in the country. Countries were included in the analysis if at least one element within the broader areas of clinical and service delivery or additional requirements to access safe abortion was addressed in an official source. However, our methodology classified a country as having information available for a category even if only one sub-question within that category was answered. For example, if a source specified who can perform abortions but provided no information on where or how they are performed, the country was still marked as having information for clinical and service delivery. As a result, we identified several countries where abortion is legal in some circumstances, but no document was found that addresses additional requirements to access safe abortion. Similarly, in other countries, no document was found that provides information on clinical and service-delivery aspects of abortion care. Thus, these numbers likely underestimate the true extent of missing information. In many other countries, only one or two sub-questions were answered, leaving significant gaps in understanding key aspects of abortion care regulation. This limited availability of comprehensive information is concerning from both human rights and public health perspectives.

Even where information exists, there is often a wide range of law and policy sources that regulate abortions in many countries. This proliferation of documents can create confusion, especially when they contain overlapping or even contradictory provisions. Moreover, this landscape adds to the burden on both abortion seekers and providers, who must interpret and reconcile multiple sources. As a result, even where abortion is legal and technically available, the complexity of navigating these sources can act as a barrier to safe and timely care.^39,34,40^

Similarly, our findings demonstrate that information on how conscientious objection is regulated is often missing. This is of particular concern, as in practice, conscientious objection may still be practised even in circumstances where it is prohibited.^41^ Where information is unavailable, it is unclear whether the law allows health workers to object to providing abortion care; if they can object, what the limits of their objections are; and whether the health system has any safeguards in place to ensure that people can access quality abortion and post-abortion care even when health workers can conscientiously object.

International human rights standards (such as observations by UN treaty bodies and special procedures) require that, where conscientious objection is permitted, states must implement safeguards.^42^ These include, for example, the obligation to ensure an effective referral so patients can access another medical professional who is willing and able to provide abortion care, and ensuring that health workers cannot invoke conscientious objection in urgent or emergency situations.^43^ Lack of publicly available information on any of these safeguards raises concerns that these safeguards do not exist, or even if they exist, are not being adequately implemented. In such circumstances, evidence suggests that crucial questions around conscientious objection are left in the hands of individual practitioners and institutions and can have serious consequences for people seeking abortions, potentially delaying or denying access to lawful services.^43,44^

### Abortion exceptionalism

Though this paper does not examine other SRH topics, the sheer volume and complexity of abortion-related legal and policy sources suggest that abortion is treated as an exception within health law and governance. This phenomenon, often referred to as abortion exceptionalism, has been widely explored by scholars,^4,8,10,45^ who note that abortion is subject to a level of scrutiny, regulation, and politicisation that far exceeds what is applied to comparable medical procedures. For example, the regulation of mifepristone has been shown to involve a disproportionately high level of restriction relative to its actual medical risks.^45^ Abortion is also treated as an exceptional issue in health education, where curricular approaches can reinforce its marginalisation.^7^ There is also evidence that countries have introduced criminal offences specifically targeting “unqualified” individuals involved in abortion, reflecting a level of legal scrutiny not typically applied to other medical procedures,^4^ and in other countries free speech jurisprudence has treated abortion-related speech differently from other forms of expression.^10^

Our findings contribute to the growing body of evidence on abortion exceptionalism by illustrating how abortion is regulated through a complex and often fragmented law and policy framework, although we did not systematically compare these patterns with the regulation of other areas of health care. In many countries, abortion-related provisions are not confined to a single legal instrument but are instead dispersed across a wide array of sources, including criminal codes, health laws, constitutions, case law, ministerial guidelines, medical ethics codes, and other legislative or regulatory documents.

This scattered legal framework is not merely structural; the nature of each source can shape how abortion is understood and practised. The fact that abortion is exceptionally regulated by a vast constellation of sources thus raises questions about how different types of regulatory instruments may influence the extent to which the legal environment supports access and respects rights. Further research could indicate whether reliance on certain sources implies a more restrictive environment or a more rights-respecting one. For example, as discussed earlier, reliance on the criminal law as a source for abortion regulation can reinforce barriers to care, including stigma and hesitancy amongst abortion seekers and providers. Similarly, the WHO has noted that regulating abortion through health regulations or clinical protocols allows for timely updates and alignment with evolving medical evidence.^1^ The role of other types of sources is more open to interrogation. Although some constitutions restrict access to abortion, others demonstrate that constitutional regulation can also establish more enabling frameworks for access.^46^ Future research is needed to assess whether and how certain types of sources – such as case law or constitutional provisions – impact the quality of abortion regulation and care.

Sources also perform a signalling function. For example, when abortion is addressed through clinical guidelines or health regulations, this indicates that aspects of abortion are viewed and regulated akin to other types of medical care.^47^ However, the use of criminal law implies that certain aspects of abortion care are viewed as comparable to serious criminal offences. Where abortion is addressed within a national Constitution, this may signal that abortion is seen as engaging fundamental rights or societal values. These varied legal framings contribute to a fragmented and often inconsistent regulatory landscape that reinforces abortion exceptionalism. Notably, this approach persists despite arguments that general health law, along with supporting regulations, professional codes, and clinical protocols, is typically sufficient to govern other areas of healthcare.^48^

Abortion exceptionalism has real-world consequences. Regulating abortion differently from other healthcare services can perpetuate misinformation, create additional barriers to access care, and reinforce stigma.^10,49^ It may, for instance, lead to the over-regulation of abortion medication, which creates barriers to access.^50^ Recognising and addressing abortion exceptionalism, both in law and in medical practice, is a critical step towards building an enabling environment for abortion care, in line with the WHO's recommendation that abortion be regulated like any other healthcare intervention. Understanding abortion exceptionalism includes understanding how it may operate in different legal and socioeconomic contexts. While this paper presents a regional categorisation of what and how many source documents states use to regulate abortion, it is also possible to analyse this across other metrics. For example, given the well-documented disparities in sexual and reproductive health access between high- and low-income countries,^51^ it is possible that the nature of abortion regulation is associated with states’ economic contexts. Although this was beyond the scope of this paper, exploring these relationships represents a valuable avenue for further study.

## Conclusions

Our findings indicate that abortion is regulated through a complex and fragmented legal framework, characterised by an over-reliance on the general criminal law, the use of multiple and sometimes contradictory sources, and information gaps in certain key areas. This regulatory complexity reflects and reinforces abortion exceptionalism. It also risks strengthening existing barriers to abortion care due to stigma, misinformation and inadequate access to information, and can have serious consequences for people's access to necessary health care and human rights.

Our findings underscore the importance of examining how the structure and clarity of regulatory frameworks can influence access to abortion care. Complex, opaque, or contradictory regulations may hinder access in practice, while gaps in regulation can result in the absence of necessary public health and human rights safeguards. These insights point to the significance of understanding not only the content of abortion laws and policies, but also the coherence and comprehensiveness of the regulatory landscape in shaping equitable access to care.

Law is a determinant of health; it can sometimes be an obstacle to ensuring progress in health and guaranteeing human rights.^2^ While further research is needed to understand the full impact of relying on the general penal code, rather than on evidence-based standards and guidelines, this approach may compound the stigma associated with the use of the criminal law in abortion regulation.^52–54^

Building on these insights, our findings contribute to the growing body of scholarship on abortion exceptionalism and the legal and practical barriers faced by those seeking and providing abortion care. They also underscore the urgent need for legal and policy reform to support an enabling environment for quality, comprehensive abortion care. Such reforms can be informed by WHO's evidence-based guidance and aim to ensure individuals can fully realise their health and human rights.
